# Eye Placement Bias Is Remarkably Robust

**DOI:** 10.1177/20416695211017564

**Published:** 2021-05-25

**Authors:** Kirsten Smith, Vera Kempe, Lara Wood

**Affiliations:** Division of Psychology, University of Abertay, Dundee, Scotland

**Keywords:** face perception, objects and features, perception, development

## Abstract

When drawing faces, people show a systematic bias of placing the eyes higher up the head than they are placed in reality. This study investigated the development of this phenomenon while removing the potential confound of drawing ability. Participants (*N* = 124) in three age groups (3–5 yo, 10–11 yo, and adults) reconstructed two foam faces: one from observation and one from memory. The high eye placement bias was remarkably robust with mean eye placement in every condition significantly higher than the original faces. The same bias was not shown for mouth placement. Eye placement was highest for the youngest participants and for the memory conditions. The results suggest that an eye placement bias is not caused by the motor skill demands required for drawing and lend evidence to the suggestion that an eye placement bias is caused by perceptual and decision-making processes.

Human eyes are positioned halfway between an individual’s chin and vertex (Farkas & Munro, 1987), which can be represented as an eye placement ratio (EPR) of around 0.5 (distance of chin to eye/distance of chin to top of head). Yet when asked to draw faces, adults (e.g., Carbon & Wirth, 2014; Ostrofsky et al., 2014), and children (McManus et al., 2012) show eye placement distortion by placing the eyes further up the head (EPRs ≃ 0.55). Furthermore, EPR distortions are more pronounced in early childhood; McManus et al. (2012) showed a trend for a developmental decline in EPR distortion from age 3 to 11, and there is developmental increase in children’s sensitivity toward distorted EPRs of less than 0.5 (i.e., the eyes are lower than halfway, De Heering & Schiltz, 2013). Thus, an eye placement bias seems most extreme in young children, showing some improvement with age, although this may be influenced by drawing ability. For example, 4 to 5 year olds make more systematic drawing errors (including orientation, simplification, and schematization) than 8 to 10 year olds (Pemberton, 1990). Understanding the role, if any, that drawing ability plays in a EPR bias is difficult because drawing is a product of perceptual, motor, and decision-making processes (Chamberlain & Wagemans, 2016). To eliminate the potential confound of developmental differences in drawing ability affecting EPR, this study moved away from the traditionally used drawing tasks in favor of a feature assembly task.

Carbon and Wirth (2014) suggest three explanations for EPR: (a) The *face-from-below* hypothesis whereby humans start life viewing human faces from below which distorts the perspective of the eye placement. (b) The *hair-as-hat* hypothesis whereby the hairline is seen as the top of the head and so eyes seem closer to the *top* than they are in reality. (c) The *head-as-box* hypothesis whereby the top of the head is seen as flat rather than convex so the drawn depiction is adjusted to represent a lower top of the head. However, Harrison et al. (2017) found that even when drawing a photo of a cat face—a face not normally seen from below, without hairline, and with a flatter top—the EPR bias persisted. Ostrofsky (2015) suggests that eye position distortion is influenced by graphic long-term memories: Eye EPRs in observation-based drawings are higher (0.55) than the average face eye placement (0.5) but lower than EPRs drawn from memory (0.6). However, it is unclear why memories of faces should be distorted, given that faces are highly familiar stimuli. Memory biases also do not explain why EPR distortions are worse in early childhood when fewer long-term memories of faces will have been formed.

A final hypothesis is that distortion of eye placement in drawings is due to perceptual eye salience leading to a placement bias. Faces are important visual stimuli with enormous social significance (Tarr & Cheng, 2003). The eyes are given a disproportionate amount of viewing attention irrespective of the species of animal being viewed, with around 50% of total viewing time spent on the eye area (Guo et al., 2010). Harrison et al. (2017) argue that when recreating stimuli, salient features are given a disproportionate amount of space within the outline of the drawn object. They found that as well as raising the position of eyes on faces, when redrawing a drawing of a house, adults raised the position of the top floor windows on the house. However, once again, drawing task demands may have influenced this bias.

The main aim of this study was to eliminate the potentially confounding task demands involved in drawing. Controlling for drawing ability will help to distinguish whether changes in EPR may, at least in part, be a product of changes in fine-motor drawing abilities across development or whether they are due to developmental differences in perception. We used simple foam faces and asked participants to reconstruct these faces either from observation or from memory. This simple design allowed us to test participants from 3 years old to adulthood. We predicted that removing the task demand of drawing would result in more accurate EPR scores in all of our samples, and that due to the simplicity of the task, there would be minimal or no EPR bias in adults reconstructing a face from observation. If the EPR bias is caused by factors relating to perception, then EPR should be higher the lower the age and should also be higher in the memory condition compared with the observational condition.

## Method

### Participants

Carbon and Wirth (2014) and Ostrofsky (2015) obtained large to very large effect sizes (*d* = ∼1.0) for their one sampled and within-subject comparisons of actual EPR. We could not obtain previously acquired effect sizes for age differences and so estimated a medium to large effect size overall. We used G*Power (Faul et al., 2009) to calculate sample size for a 3 (Age) × 2 Repeated Measures (Memory vs. Observation) analysis of variance (*f* = 0.325, a err prob = 0.05, recommended sample size =114).

Participants (*N* = 124) were recruited through convenience sampling in Central Scotland, UK. There were 39 participants aged 3 to 5 (16 males), 45 participants aged 10 to 11 (23 males), and 40 participants that were over 18 years old (eight males). All participants completed both observation-based and memory-based face recreation. No participants were excluded. Consent for child participation was gained from a primary caregiver and the child. All data were anonymized from the point of collection.

### Design

This was a 3 × 2 design with participant age as a between-subject variable (three levels: 3–5, 10–11, and adult) and face recreation as a within-subjects variable (two levels: observation vs. memory). The order of recreation based on observation versus memory was systematically counterbalanced as was face type (male vs. female).

### Materials

**Figure 1.**
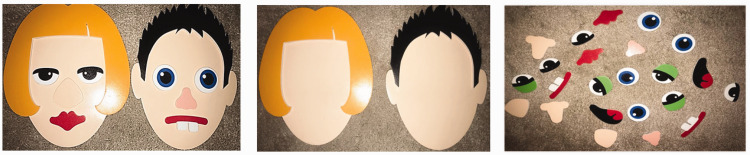
Female and male model face stimuli (left panel), blank faces (middle panel), and facial features (right panel).

This study used foam faces and facial feature from two sets of *Meadow Kids Silly Faces Bath Stickers*. Onto two identical foam faces, cut-out pieces of hair, eyes, nose, and mouth were affixed with polyvinyl acetate (PVA) glue, creating a *male* and *female* face (see Figure 1). The eyes in both models were placed at an identical mid-point between the chin and the head crown of 116 mm from the middle of the tear ducts to the chin, creating an EPR of 0.5. A further two blank foam faces, one for each model gender, were used for recreation. The blank faces only had hair that was identical to the hair of the same-gender target face. Alongside the blank faces, there were different facial features, four blue eyes, four green eyes, four black eyes, two square noses, two small noses, two large noses, two closed lips, two lips with teeth, and two lips with a tongue. A digital still camera was used to take a picture of the faces once a participant had completed testing. In addition, trace paper was used with a ruler and a pencil to measure and record the distance between the middle point of the tear ducts and the chin on the foam faces to measure eye position.

### Procedure

Participants sat at a table and were asked to look at the model foam face (male or female). In front of them was a corresponding blank template face and an array of facial features. For the memory condition, individuals were shown the model face for 10 seconds before it was removed from sight. In the observation condition, participants were shown the model face during the entire recreation process. Participants were asked to try to assemble the model face using the available facial features. All participants were given 60 seconds to complete the face.

## Results

### Coding

To determine eye placement, the line between the tear ducts as well as the lowest point of the chin were marked on trace paper, and the distance between the mid-point of this line and the marked chin point was measured in mm and converted to an EPR ratio. To determine accuracy of feature recollection, photographs of each recreation were coded with respect to correct features; one mark was given for each feature that matched the model: eyes, nose, and mouth.

Additional posthypothesis analysis investigated mouth placement. Mouth placement ratio (MPR) was obtained by measuring the distance from chin to a precise and equal point on each model’s mouth (MPR = 0.21) and dividing it by the total height of the face. Measurements were obtained from the photographs taken to record feature recall (see “Coding” section). Measurements could not be obtained on 22 faces because six photos did not include the chin, 12 mouths were placed upside down, 3 faces had the wrong mouth, and 1 mouth was placed above the nose.

### Analyses 

Accuracy of feature recall data did not meet parametric assumptions and the number of facial features correctly recalled was near ceiling with a median of three (out of three) for all age groups, in both the observational and memory conditions. However, there was a main effect of face recreation and age on recall; feature recall was poorer in the memory compared with the observation condition (Wilcoxon signed-ranks test, *Z* = −3.41, *p* = .001). Overall, 3 to 5 year olds recalled fewer correct features than 10 to 11 year olds (observation Mann–Whitney *U* = 758, *p* = .046, memory *U* = 576, *p* < .001) and adults (observation *U* = 640, *p* = .005, memory *U* = 588, *p* = .014); there was no significant difference in recall accuracy between 10 to 11 year olds and adults; (observation *U* = 860, *p* = .18, memory *U* = 805, *p* = .098).

The main analyses focused on the placement of the eyes relative to the model face EPR of 0.5. We first checked whether, as expected, adult participants in the observational condition showed minimal or no EPR bias when recreating the face. A one-sample *t* test showed a significant difference between the adult’s observational EPR (*M* = 0.61, 95% confidence interval [CI] [0.60, 0.63]) and the 0.5 EPR of the models, *t*(39) = 15.0, *p* < .001, Cohen’s *d* = 1.96. The remaining five conditions also showed this bias (all conditions had a mean EPR greater than 0.61 and were significantly different from the 0.5 model EPR; one sample *t* tests *p*s < .001, all Cohen’s ds > 1.78).

**Figure 2.**
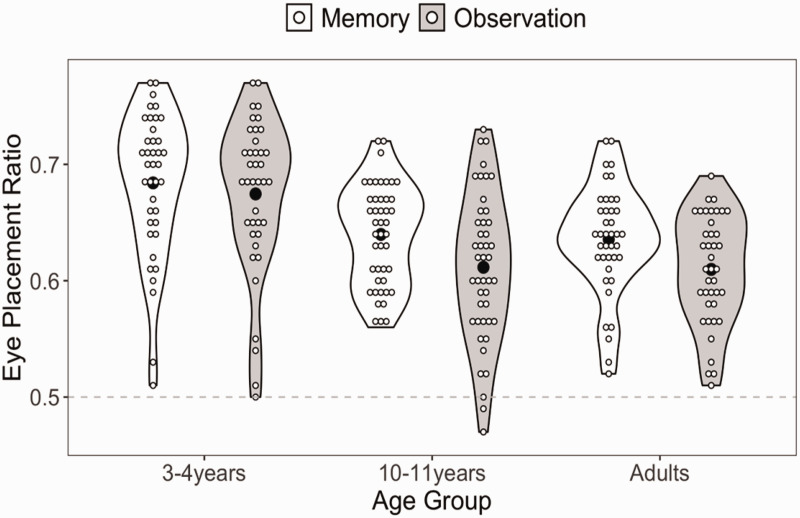
Violin plot showing all data points (white circles) as well the group means (black circles). The dashed line indicates the O.5 EPR present in both models.

A general linear model was fitted to investigate the effects of age and face recreation on EPR distortion (Figure 2). The main effect of face recreation was significant, *F*(1, 121) = 24.36, *p* < .001, partial η^2^ = .17. EPR in the observation condition was significantly lower (*M* = .63, 95% CI [.62, .64]) than EPR in the memory condition (*M* = .65, 95% CI [.64, .66]). The main effect of age was significant, *F*(2, 121) = 16.50, *p* < .001, partial η^2^ = .21. Post hoc Bonferroni tests showed that in the observation condition, the 3 to 5 years olds (*M* = .67, 95% CI [0.65, 0.70]) placed the eyes significantly higher than the 10 to 11 year olds (*M* = .61, 95% CI [.59 and .63], *p* < .001) and the adults (*M* = .61, 95% CI = [.60, .63], *p* < .001). There was no significant difference between the 10 to 11 year olds and the adults (*p* > .99). Likewise, in the memory condition, the 3 to 5 year olds (*M* = .68, 95% CI = [.66, .70]) placed the eyes significantly higher than the 10 to 11 year olds (*M* = .63, 95% CI *=* [.63, .65], *p* < .001) and the adults (*M* = .64, 95% CI = [.62, .65], *p* < .001). There was no significant difference between the 10 to 11 year olds and the adults (*p* = .97). The interaction between age and face recreation was not significant, *F*(2, 121) = 1.83, *p* = .166.

Additional analysis investigated whether EPR was a product of a general bias to give more space to salient objects in which case it should also have affected the placement of the mouth. Unlike EPR, where every participant in both conditions placed the eyes *higher* than the model, mouths were placed above (48.4% had MPR > 0.21), below (38.5% had MPR < 0.21 = 38.5%) and equal to (13.3% had MPR = 0.21) the placement of the model’s mouth. One-sample *t* tests showed no significant difference between the model MPR of 0.21 and observational MPR, *M* = 0.21, 95% CI [0.21, 0.22], *t*(111) = 0.77, *p* = .44, or memory MPR, *M* = 0.21, 95% CI [0.21, 0.22], *t*(113) = 1.06, *p* = .29. We also investigated whether the face type (male or female with differing eye and hair features) had an effect. There was no significant difference between EPRs for the male versus the female faces in either the memory, *t*(122) = −0.28, *p* = .779, or observation condition, *t*(122) = 0.50, *p* = .617.

## Discussion

 Previous research had identified an eye placement ratio (EPR) bias of around 0.55 which is a deviation from the correct EPR of 0.5 (Carbon & Wirth, 2014; McManus et al., 2012; Ostrofsky et al., 2014). We predicted that, when drawing abilities were controlled for using a simple reconstruction task, EPR bias would be reduced in all groups. There was no such reduction, with participants positioning the eyes with an EPR of around 0.6. The prediction that children would exhibit a stronger EPR bias than adults, because EPR is a product of perceptual biases that changes across development, was partially supported; 3 to 4 year olds showed poorer performance as predicted, but 10 to 11 year olds did not differ much from adults. The results suggest that an EPR bias is not caused by drawing abilities, and lend evidence to the suggestion that an EPR bias is caused by perceptual and decision-making processes.

From a perceptual point of view, eyes may be subject to several principles of perceptual grouping. One such principle is the Gestalt principle of form similarity, which postulates that among equidistant stimuli those who share salient features, such as shape, tend to be grouped together and the distance to the surrounding stimuli is systematically overestimated (Wertheimer,1923/1958). With respect to facial features, perceptual grouping by similarity can account for the EPR bias because the two eyes are more similar to each other than each eye is to the nose or mouth. As a result, systematic overestimation of the distance to the nose would then move the eyes higher up in the face. Grouping by similarity is considered a low-level, pre-attentive process (Treisman, 1982) which emerges as early as 6 to 7 months of age (Quinn et al., 2002). Perceptual distortion of distance due to similarity-based grouping occurs in perceptual estimations, reconstructions, and drawings, and it shows a developmental trajectory similar to the one documented here, with greatest distortions in 4-year-old children and minimal differences between 8 year olds and adults (Farran & Cole, 2008). Such perceptual grouping may extend to aesthetic preferences for placement of objects surrounded by more white space (Liu & Ko, 2017) and may therefore also account for elevated placement of eyes and windows in drawings of cat faces and houses, respectively (Harrison et al., 2017). However, perceptual grouping has typically been demonstrated for geometric shapes such as squares, lines, or circles.

Another perceptual grouping principle that may contribute to perceptual salience of the eyes is symmetry (Machilsen et al., 2009). Grouping by symmetry tends to be particularly robust when it involves mirroring along the vertical axis (e.g., Kahn & Foster, 1986), as is the case for the eyes. As a ubiquitous feature of many organisms, it appears to facilitate figure-ground segmentation and pattern detection in noise, perhaps owing to the adaptive value of regularity detection (Tyler, 1996). While determining the precise perceptual grouping mechanisms that may affect eye prominence is beyond the scope of this study, the persistent increased EPR suggests that perceptual grouping of eyes occurs despite the fact that faces are ubiquitous in daily human life and configurations of facial features are higher in familiarity than arrangements of geometrical objects.

One possibility for why the distorting effect of grouping the eyes together is so persistent that it cannot be overridden by direct observation and familiarity is that the eyes carry a significant amount of social information. Specifically, humans rely on gaze following as a major cue toward an interlocutors’ locus of attention (Langton et al., 2000) which enables joint attention as a foundation for the development of communicative abilities (Tomasello et al., 2007). Although mouths also display salient emotional cues, the eyes are considered to be more reliable indicators of certain emotional states such as anger, fear, and sadness (Bombari et al., 2013). Thus, the perceptual salience of the eyes may be reinforced because the ability to read others’ mental states is crucial for humans to navigate their complex social world. Indeed, infants show an increasing bias in attending to the eyes as opposed to all other facial areas (Haith et al., 1977), and this bias extends into adulthood (Hunnius et al., 2011). Accordingly, the ability to glean mental states from the eyes shows a steady increase across childhood into adulthood (Phillips et al., 2002; Tonks et al., 2007).

We had predicted that removing motor demands associated with drawing might reduce the EPR. Instead, we found a larger EPR than drawing tasks, even in adults, suggesting that the bias may have been exacerbated by our task. One possible explanation for this finding is that drawing may afford additional means of increasing the visual salience of the eyes, such as increasing their size, that is, removed when faces are assembled from ready-made parts. In the absence of these additional means exacerbating the EPR may have provided a compensatory mechanism that serves to preserve the visual representation of their social significance. This conjecture could be tested in future research on face drawings by measuring increases in eye size and contrast.

Despite the social significance of eyes, faces are processed configurally, which includes sensitivity to first-order relations of facial features and the distances among features (McManus et al., 2012). However, this configural face processing is slow to develop (Mondloch et al., 2002) which may explain why EPR due to low-level perceptual grouping of the eyes is compounded by greater reliance on featural face processing in young children. For adults, one would expect that the sensitivity to distances between facial features that comes with configural processing might protect from perceptual distortion. However, research has shown that configurational processing of faces is contingent upon presence of the eyes attesting to their special role in face perception (Itier et al., 2007). It would appear that our persistent inability to reconstruct faces correctly may be the small price we pay for perceptual predispositions that have adapted to the extraordinary significance of eyes as social signals.
